# Evaluating the Endophytic Activities of *Beauveria bassiana* on the Physiology, Growth, and Antioxidant Activities of Extracts of Lettuce (*Lactuca sativa* L.)

**DOI:** 10.3390/plants10061178

**Published:** 2021-06-09

**Authors:** Neo Macuphe, Oluwafemi Omoniyi Oguntibeju, Felix Nchu

**Affiliations:** 1 Department of Horticultural Sciences, Cape Peninsula University of Technology, P.O. Box 1906, Bellville 7535, South Africa; neomacuphe@gmail.com; 2 Phytomedicine and Phytochemistry Research Group, Oxidative Stress Research Centre, Department of Biomedical Sciences, Faculty of Health and Wellness Sciences, Cape Peninsula University of Technology, P.O. Box 1906, Bellville 7535, South Africa; oguntibejuo@cput.ac.za

**Keywords:** *Myzus persicae*, *Beauveria bassiana*, *Lactuca sativa*, endophytic, colonization

## Abstract

Endophytic entomopathogens have growth promoting, nutrient fortifying, and anti-insect properties that could improve the yield and quality of lettuce (*Lactuca sativa* L.). *Lactuca sativa* is a vegetable crop with high demand; however, it is susceptible to aphid infestations. This study’s objectives were to assess the pathogenicity of *Beauveria bassiana* (strain: SM3) (Bals.) Vuil. (Hypocreales) against *Myzus persicae* Sulzer, tissue colonization of lettuce by conidia of *B. bassiana*, as well as the effects of fungal inoculation on growth, tissue nutrient content, and proximate composition of the lettuce plants. Furthermore, the involvement of tissue nutrients in mediating the influence of endophytic fungus on the plant traits was examined. Insects and plants were exposed to four fungal conidial concentrations: 0, 1 × 10^6^, 1 × 10^7^ and 1 × 10^8^ conidia mL^−1^ in an anti-insect bioassay and a greenhouse experiment, respectively. The *B. bassiana* strain was pathogenic against *M. persicae*, inducing mean insect mortality of 78% at the highest concentration (1 × 10^8^ conidia mL^−1^). The *B. bassiana* endophytically colonized up to 76% of plants exposed to 1 × 10^8^ conidia mL^−1^. Crown size and plant height varied significantly among treatments. However, the plant fresh and dry weights and nutrient elements N, P, K, Ca, and Mg did not vary significantly among treatments. Among the plant macronutrients assessed, only tissue carbon content was significantly (*p* < 0.01) affected by conidial treatments. The tissue C and Cu contents significantly correlated with the antioxidant capacity of the lettuce plants. Most of the micronutrients, viz. Mn, Fe, Cu, and B were remarkably higher (*p* < 0.05) in the fungus-treated plants than in the control plants. The antioxidant capacity (FRAP and TEAC) of plant extracts varied significantly (*p* < 0.001) among treatments, with the highest conidial treatment yielding the most increased antioxidant activity. In conclusion, the *B. bassiana* strain was endophytic to lettuce, pathogenic against *M. persicae*, and induced increased micro-nutrient tissue contents and antioxidant activities. This study demonstrated that *B. bassiana* could be potentially used in the biofortification of nutritive and medicinal qualities of plants.

## 1. Introduction

*Lactuca* (Asteraceae) is one of the most consumed salad vegetables in North America, South America, Europe, Australia, and New Zealand. Aphid infestations on this crop often lead to declines in lettuce yields and economic losses among commercial and small-scale farmers [1]. Aphids are sap-feeding insects that are capable of causing direct injury to plants and vectoring many damaging phytopathogens, including the lettuce mosaic virus [1]. Reducing aphid infestations is an efficient strategy to manage viruses and other vector-borne phytopathogens [2]. However, conventional aphid control still depends mostly on synthetic insecticides, which are often toxic to the environment [3,4]. Furthermore, prolonged, excessive, and widespread use of these chemicals have been associated with insect resistance to insecticide and reduced soil fertility [5]. Benign measures of aphid control need to be developed to achieve high quality and yield of lettuce. 

Endophytic entomopathogenic fungi can colonize plant tissues without causing damage or disease to host plants [6]. They have been found in the tissues of different crops. Endophytic entomopathogenic fungi are vital in the agricultural and horticultural sectors because they provide protection against herbivorous insects through systemic resistance and are compatible with systemic insecticide application [7,8]. The use of fungal entomopathogens as endophytes in biological control has gained interest among researchers since 2010, resulting in a marked increase of published papers on the topic [9]. Endophytic fungi can enhance plant growth directly or indirectly [10] and improve plant adaptation to adverse conditions, including biotic and abiotic stresses [11,12,13,14].

Researchers are interested in the mechanisms through which endophytic fungi, including endophytic entomopathogenic fungi, protect plants and improve plant yield and nutritive value. For example, *Beauveria bassiana* (Bals.) Vuil. (Ascomycota: Hypocreales) can help plants adapt to different conditions, facilitate the transfer of nutrients from the soil to the roots, and regulate plant growth and defense [15,16,17]. Some endophytic fungi act as bio-stimulants that help disseminate macronutrients and micronutrients [18]. Nutrient translocation and uptake can enhance plant growth by modifying phytohormones [14,19]. Endophytic fungi and bacteria can produce or assist plants in producing secondary metabolites that can defend the plants against pathogens and pests [17,20,21]. White Jr and Torres [20] reported that plants colonized by endophytes produce more glucose and fructose. Some studies suggest that endophytic fungi stimulate antioxidants production in plants, which is essential for neutralizing reactive oxygen species [22]. Fungal endophytes can also solubilize phosphate [23] and produce phytohormones such as cytokinins, indole acetic acid (IAA), gibberellin (GAs), and siderophore. Some fungi supply essential vitamins to the plant host [12,24,25,26,27,28]. A study by Hamayun et al. [29] suggested that endophytic fungi enhanced proximate composition in plants. Endophytic entomopathogenic fungi have the potential to provide solutions to agricultural and medical problems [30].

One of the most studied entomopathogenic fungi is *Beaveria bassiana* (Bals.) Vuil. (Ascomycota: Hypocreales). Besides being a prolific insect pathogenic fungus, it is naturally endophytic in many crops [31]. Its endophytic trait is being exploited in the field of agriculture and medicine. *Beauveria bassiana* is a soil-borne cosmopolitan fungus, having a very wide range of hosts and is the basis for the production of many bioinsecticides against a wide range of insect pests [32]. Many isolates of *B. bassiana* have been used to artificially colonize tissues of food crops, including cucumber [31], tomatoes [33] and cabbage [34]. However, information on the endophytic activity of *B. bassiana* on lettuce is lacking; hence, the motivation for this study.

Although many studies have examined the effects of endophytic fungi on plant growth and secondary metabolites, few studies have simultaneously and comprehensively investigated the effects on tissue colonization, plant growth, nutrient contents, antioxidant capacity, and proximate components. Among these plant traits, tissue nutrient (mineral) content is central because it can significantly influence the other plant traits. For example, at tolerable ranges, higher levels of tissue macronutrients correlate with higher plant growth [35], higher chlorophyll contents [36] and lower secondary metabolites and antioxidant capacity [37,38].

A few studies have investigated the mechanisms through which nutrient elements mediate fungal endophytes’ influences on plant traits. Recently, Chen et al. [39] demonstrated that increased concentration of many nutrient elements in leaves and roots is one of the possible mechanisms by which endophytic fungus *Epichloë festucae* var. *lolii* enhances survival of *Lolium perenne* in low fertility soil. Exposing *L. sativa* to Ca ions that were encapsulated in biopolymeric microparticles had a positive effect on secondary metabolites, antioxidant activity and chlorophylls [40]. *Piriformospora indica* stimulated both plant growth and P acquisition of trifoliate orange [41]. This study addresses two research questions. What are the effects of *B. bassiana* inoculation on the growth, physiology and the nutritive value of lettuce? Do tissue nutrients mediate *B. bassiana*’s endophytic influence on the plant’s traits? It is necessary to optimize the application of endophytic entomopathogenic fungi in biofortification, pest management and medicinal plant cultivation projects. More studies are needed to fully decipher how tissue minerals mediate the effects of endophytic fungi on plant functional traits, such as biomass, antioxidants, and proximate composition.

This study is intended to provide a deeper understanding of the effects of the endophytic entomopathogenic fungus *B. bassiana* (strain: SM3) on the physiology and nutritive value of *L. sativa*. The specific objectives of this paper were: (i) to assess the pathogenicity *of B. bassiana* against *M. persicae* in the laboratory, (ii) to assess colonization of lettuce by *B. bassiana*, (iii) to assess the effect of *B. bassiana* on growth, macronutrient and micronutrient contents, antioxidant contents, proximate components of *L. sativa*, chlorophyll content of lettuce, and (v) to explore the mediating role of tissue nutrient on the influence of endophytic *B. bassiana* on lettuce traits.

## 2. Results

### 2.1. Pathogenicity Assessment

Generally, the results showed that *B. bassiana* (strain: SM3) was pathogenic against *M. persicae.* Insect mortality increased with conidial concentration. The highest concentration (1 × 10^8^ conidia mL^−1^) caused higher insect mortality (78%) compared to other treatments (DF = 3, 20; F = 21.57; *p* < 0.01) (Table 1). The isolate had LC_50_ value of 1.1 to 1.6 × 10^6^ conidia mL^−1^ (Table 1).

### 2.2. Colonization of Tissues by Fungus

**Figure 1:**
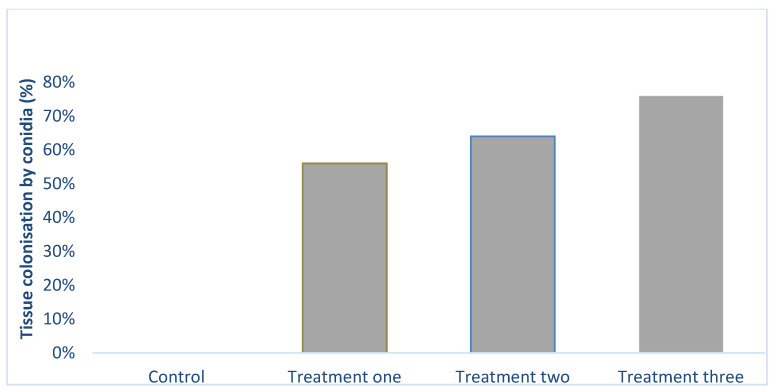
The fungal outgrowth on leaf section of *L. sativa* following *B. bassiana* inoculation at different conidia concentrations (Control = 0, treatment one = 1 × 10^6^ conidia mL^−1^, 1 × 10^7^conidia mL^−1^ and 1 × 10^8^ conidia mL^−1^) (*n* = 100).

*Beauveria bassiana* was successfully re-isolated from the leaves of the plants at three weeks post-treatment. The number of plants colonized by the fungus increased significantly (X^2^ = 34.05; DF = 3; *p* < 0.001) with fungal concentration. The highest tissue colonization of 76% (Figure 1 and Figure S1) was obtained at 1 × 10^8^ conidia mL^−1^ followed by treatments two (1 × 10^7^) and one (1 × 10^6^), 64% and 56%. Similar results were obtained with the roots. The leaf and root sections from the control treatment had no fungal outgrowth.

### 2.3. Effect of Fungus on Plant Height, Crown Size and Roots Length

Generally, the *B. bassiana* inoculation significantly increased plant height (DF = 3, 196; F = 3.61 *p* < 0.01); the heights ranged from 15.52 to 16.04 ± 0.18 (Table 2). Similarly, there was a significant difference among the treatments in terms of crown size of the plant (DF = 3, 196; F = 14.52; *p* < 0.001); generally, fungus-treated plants had larger crown sizes (Table 2). Unlike plant height and crown size, there was no significant difference in root lengths (DF = 3, 116; F = 0.996; *p* = 0.40).

#### Effect of Fungus on Fresh Weight and Dry Weight of the Root and Aerial Parts of Lettuce Plants

The fungus inoculation did not significantly affect (DF = 3, 116; *p* > 0.05) the fresh and dry weights of the root and aerial parts (Table 3).

### 2.4. Effect of Fungus (Beauveria bassiana) on Antioxidant Capacity: Ferric Reducing Antioxidant Power (FRAP) and Trolox Equivalent Antioxidant Capacity (TEAC)

The treatment significantly influenced antioxidant capacity in plant extracts (DF = 3, 8; F = 6.067 *p* < 0.001 FRAP _(µmol AAE/g)_; DF = 3, 8; F = 31.669; *p* < 0.001 TEAC **_(µmol TE/g_**), with higher levels occurring in the plants inoculated with the highest fungal conidial concentration (1 × 10^8^ conidia mL^−1^) and in control treatments (Table 4) compared to the lower conidial concentrations of 1 × 10^6^ conidia mL^−1^ and 1 × 10^7^ conidia mL^−1^. The association between C content and antioxidant capacity was significant; TEAC (R^2^ = 0.87; y = 1.379x −462.93) and FRAP (R^2^ = 0.88; y = 0.918x −282.3) (Table S1). Among the tissue micronutrients, only tissue Cu level was strongly association with the antioxidant capacity; FRAP (R^2^ = 0.741; y = −0.05x + 8.11) and TEAC (R² = 0.75; y = −0.036x + 6.71) (Table S1).

### 2.5. Effect of Beauveria bassiana on Tissue Analysis

#### 2.5.1. Macronutrients

Among all the macronutrients, only carbon (C) tissue content was significantly (DF = 3, 8; F = 34.67; *p* < 0.01) influenced by fungal treatment. Carbon varied significantly with fungal treatments; plants in the control and highest conidial concentration had relatively higher tissue carbon contents than the moderate conidial treatments (1 × 10^6^ conidia mL^−1^ and 1 × 10^7^ conidia mL^−1^). No significant differences in N, P, K, Ca, and Mg tissue contents were found among treatments (Table 5).

#### 2.5.2. Micronutrients

Unlike the macronutrients, most micronutrients were significantly affected by conidial concentration in this study. Apart from Zn, all the other tissue micronutrients (Mn, Fe, B, Cu) assessed varied significantly (DF = 3, 8; *p* < 0.05) among treatments, with a discernible association of fungal treatments and higher plant tissue micro-nutrient contents. The highest values for Mn (81.03 ± 4.39), Cu (5.90 ± 0.26), and B (50.27 ± 1.01) were observed at 1x 10^7^ conidia mL^−1^ (Table 6). The tissue Fe (iron) content was highest at the highest conidial treatment, lowest in the control treatment, and the differences among treatments were statistically significant (DF = 3, 8; F = 7.956; *p* < 0.01).

### 2.6. Proximate Results

#### 2.6.1. Protein

Generally, there was a significant difference in protein contents among treatments (DF = 3, 8; F = 5.18; *p* < 0.05). Plants in the treatment 1× 10^6^ conidia mL^−1^ had the lowest protein content compared with the other treatments (Table 7).

#### 2.6.2. Fatty Acids

Fungal inoculation had no influence on palmitic acid (DF = 3, 8; F = 0.66 *p* > 0.05), linoleic acid (DF = 3, 8; F = 4.00; *p* > 0.05), linolenic acid (DF = 3, 8; F = 3.14; *p* > 0.05) and total fatty acids (DF = 3, 8; F = 1.17; *p* > 0.05). However, the total fatty acids were relatively higher in the control plants (1566.67 ± 233.33) than in those of fungal treatments (Table 8).

### 2.7. Chlorophyll

Generally, the exposure to *B. bassiana* conidia did not statistically affect chlorophyll contents (DF = 3, 8; F = 2.45; *p* > 0.05; total chlorophyll µg g^−1^), (DF = 3, 8; F = 2.26; *p* = 0.05; chlorophyll a µg g^−1^) and (DF = 3, 8; F=2.96; *p* = 0.05; chlorophyll b µg g^−1^). However, the chlorophyll contents were higher at 1 × 10^8^ conidia mL^−1^ (Table 9).

## 3. Discussion

The *B. bassiana* strain used in this study was pathogenic against female aphids and colonized lettuce plants in the laboratory and greenhouse experiments, respectively. Despite the evidence of successful tissue colonization by the fungus, minimal effects were observed on tissue macronutrient contents. However, conidial inoculation significantly (*p* < 0.05) enhanced tissue micronutrient, carbon tissue, and antioxidant contents. Fungal inoculation had varied effects on the different growth parameters. 

The *B. bassiana* used in this study induced aphid mean mortalities ranging from 48–78% insects in the *in vitro* bioassay, increasing significantly (*p* < 0.001) with concentrations and demonstrating that this fungus is pathogenic against aphids [42,43,44]. *Beauveria bassiana* species has been known to be particularly virulent against sap-sucking homopterans. It can secrete mycotoxins, including proteases and chitinases, which degrade insect cuticles [45].

This study demonstrated that, depending on the concentration, the *B. bassiana* (strain: SM3) colonized 56% to 76% of lettuce’s leaf tissue at six weeks post-treatment. The fungal colonization observed in this study could be described as moderate to high. The colonization of plant tissues by a fungus can be influenced by several factors, including the concentration of fungal conidia, age of the plant [46], and type of fungal strain selected [47,48]. Moreover, some inoculation methods enhance colonization of plant tissues by endophytic entomopathogenic fungi [49]. For example, seed inoculation produced higher colonization than seedling inoculation in onion plants [49].

The effects of *B. bassiana* isolate used in this study varied according to the plant growth parameter. Plant height and crown size of the plants were significantly affected (*p* < 0.05) by fungal inoculation, while fresh and dry weights were not significantly affected (*p* > 0.05) despite fungal colonization of the lettuce. Nevertheless, it is widely believed that fungal endophytes promote plant growth [50,51]. Previously, Dash et al. [52] reported that entomopathogenic fungi such as *B. bassiana*, *Isaria fumosorosea* and *Lecanicillium lecanii* enhanced the growth of bean plants. However, the effect of endophytic fungi on plant growth parameters can vary with host and isolates [48]. The mechanisms through which the endophytic fungi influence the different growth parameters need to be better understood. Endophytic fungi produce siderophores and organic acids, altering the bioavailability of a variety of nutrients [52].

Despite the successful colonization of plant tissues, there was no statistical difference in tissue macronutrients for most macronutrients measured, i.e., N, P, K, Ca, and Mg. Because N, P, K, Ca, and Mg are important for increased plant growth and biomass accumulation and were not affected by *B. bassiana* inoculation in this study, it is, therefore, not surprising the results in the current study showed that the fungus had little effect on plant fresh and dry weights. Similarly, the fungus did not influence the chlorophyll content. N, Ca, K, and Mg are key nutrients that are responsible for the synthesis of chlorophyll. Previously, Rozpądek et al. [53] demonstrated that endophytic fungus improves chlorophyll content in plants. However, the fungus affected C tissue content; it was higher in the control plants and the highest concentration of 1 × 10^8^ conidial mL^−1^ compared with plants in the other treatments (1 × 10^6^ and 1 × 10^7^) (Table 5). The high carbon content might be linked to higher amounts of structural carbon or carbon-based compounds [54] or increased carbohydrate accumulation due to increased photosynthesis [55,56]. Moreover, some microbes have high-affinity transporters that enable them to detect and absorb organic carbon nutrients and minerals in the form of organic acid-metal complexes from a growth medium or soil [57]. However, the reason for the lower tissue carbon in treatments 1 × 10^6^ and 1 × 10^7^ conidia mL^-1^ in this study is not clear. It is necessary to carry out further studies to understand the relationship between endophytic fungi and carbon content in plants. 

The trend of the tissue micronutrient contents was quite obvious and interesting, as shown in the current results. There were statistical differences (*p* < 0.05) between fungal treated and control plants in four of the five micronutrients assessed, with control plants having consistently lower levels of Mn, Fe, Cu, and B than fungus-inoculated plants. Endophytic fungi can synthesize some of these micronutrients and improve the uptake of these nutrients [58]. While these elements are needed in small quantities, they have important physiological roles in plants: Fe is needed for the biosynthesis of chlorophyll in plants [59]; B is an essential microelement in the metabolism of nucleic acid, carbohydrates and protein [60]; Cu plays critical roles in the physiological processes of plant such as photosynthesis, respiration, carbohydrate distribution, nitrogen fixation, metabolism of protein and antioxidant activity [61] and Mn is significant for metabolism and plant development [62]. The deficiency or excess of these micronutrients can be detrimental to plants [63,64]. For example, Fe deficiency can cause yellowing and chlorosis on new leaves and reduces sugar metabolic enzymes [65]. Furthermore, the production of secondary metabolites is influenced by the concentration of micronutrients in plants, and these micronutrients are needed in small amounts [63,64]. In this study, we observed that plants that were exposed to the highest concentration of fungal suspensions yielded the highest Fe content and antioxidant capacity. On further examination, we found weak correlation between Fe content and antioxidant capacity, TEAC (R^2^ = 0.0966; y = 0.1275x + 116.94) and FRAP (R^2^ = 0.01; y=0.0975x + 108.69) despite Fe being a powerful reducing agent ([66,67]. Nevertheless, Cu content significantly correlated with antioxidant capacity, FRAP (R^2^ = 0.741; y = −0.05x + 8.11) and TEAC (R² = 0.75; y = −0.036x + 6.71) (Table S1).

Another interesting observation was the strong correlation between C content and antioxidant capacity; TEAC (R^2^ = 0.87; y = 1.379x − 462.93) and FRAP (R^2^ = 0.88; y = 0.918x − 282.3) assays. It is worth noting that carbon-based secondary metabolites have antioxidant properties [68], and some endophytic fungal strains are a source of antioxidants [69]. Proximate and phytochemical analyses may help clarify the nature of the carbon in the plant tissue. Future studies on metabolomics may also help explain the relationship between the carbon contents and the antioxidant capacity.

Endophytic fungi are capable of enhancing or producing biochemical metabolites that can be exploited in agriculture [70,71]. Based on the proximate analysis protein was significantly different (*p* < 0.05). This is interesting because the tissue nitrogen content, which often correlates with protein content, was not significantly (*p* > 0.05) affected by *B. bassiana* inoculation. Further investigations are needed to determine the protein types and sources. Proteins play an important role in the growth and nutritive value of plants and can mediate the production of antioxidants [72]. The fatty acids’ contents did not vary significantly (*p* > 0.05) among conidial concentrations.

## 4. Materials and Methods

### 4.1. Research Design

Laboratory and greenhouse experiments were carried out in this study. The selected *Beauveria bassiana* (SM3) was evaluated using an insect mortality bioassay to determine if it was pathogenic *Myzus persicae* and could be considered for further study—the greenhouse study. In the greenhouse study, potted lettuce plants were allocated to one of four treatment groups in a complete randomized design, with a single factor. Plants in each treatment group were exposed to one of four fungal conidial concentrations: 0, 1 × 10^6^, 1 × 10^7^, and 1 × 10^8^ conidia mL^−1^. The effects of fungal inoculation on plant growth, plant physiology, plant secondary metabolites, and insect infestations were assessed. All experimental plants were maintained under the same environmental conditions.

### 4.2. Plants Material

Lettuce (L. *sativa*) seedlings (cultivar: Green Oak) were sourced from Stodels Nurseries (Pty) Ltd in Bellville, Western Cape Province, South Africa. They were kept in the greenhouse of the Cape Peninsula University of Technology (CPUT), Bellville, South Africa, under the following conditions: 25 ± 2 °C, 60–80% RH, and 14/10 natural light/dark regime. Each plant was gently removed from the six-pack tray of lettuce seedlings and transplanted into a 15 cm diameter pot containing a substrate mix: one-part silica sand, one-part perlite, and one-part peat moss. Before its use, the substrate mix was sterilized with 1% sodium hypochlorite for 30 min and was rinsed with sterile distilled water three times. Plants were fed using recommended hydroponics Nutrifeed^®^ hydroponic fertilizer (Starke Ayres Pty. Ltd., Cape Town, South Africa). The fertilizer was mixed with sterile distilled water at a concentration of 10 g/5000 mL, and each potted plant was drenched with 200 mL once a week. Subsequently, each plant was watered with distilled water once a week for six weeks.

### 4.3. Fungus Preparation

An existing *B*. *bassiana* (SM3) strain that was previously isolated from a vineyard and identified molecularly by Moloinyane and Nchu [48] was used in this study. The fungus was cultured on a selective medium: half-strength (19.5 g/1000 mL) of Potato Dextrose Agar (PDA) (Sigma-Aldrich PTY. LTD., Johannesburg, South Africa), 0.04 g streptomycin, and 0.02 g ampicillin sodium salt. The PDA was prepared in 9 cm- and 14 cm-diameter Petri dishes. Fungal cultures were incubated for three weeks at 25 ± 2 °C in the darkness. The mature conidia of *B. bassiana* were harvested using a sterile spatula and transferred into a 50 mL centrifuge tube containing 30 mL sterile water. The tube was capped and shaken for 3 min and mixed vigorously for two minutes using a vortex mixer (MI0101002D Vortex Mixer, Silverson Machines, Inc., East Longmeadow, MA, USA) at 3000 rpm to homogenize the conidial suspension. The homogenous conidial suspension was transferred into 1000 mL bottles containing 500 mL sterile distilled water and 0.05% Tween 80 (Polysorbate, Sigma-Aldrich, Johannesburg, South Africa). The conidia concentration was determined using a haemocytometer (Bright-Line, Sigma-Aldrich, Johannesburg, South Africa) and observed with a light microscope at 400× magnification to determine the required concentration of (1 × 10^8^, 1 × 10^7^, and 1 × 10^6^ conidia mL^−1^). Germination percentage was assessed on 100 spore count at 40× magnification [73]. Each plate was replicated four times, and over 90% germination was observed.

### 4.4. Pathogenicity Against Myzus Persicae

The pathogenicity of *B. bassiana* strain (SM3) on *Myzus persicae* was tested against the three different conidial suspensions of endophytic fungus *B. bassiana* (SM3). The leaf dip method was adopted for pathogenicity bioassay. Three conidial concentrations and control were used to determine the virulence of the fungus against the aphid. The spores were adjusted into three concentrations (1 × 10^8^, 1 × 10^7^, and 1 × 10^6^ conidia mL^−1^), and the control had only 0.05% Tween 80 and sterile water. For each fungal treatment, a lettuce leaf section (with a diameter of 50 mm) was cut and immersed into 5 mL conidia suspension for 10 s. While for the control treatment, the leaf section (diameter of 50 mm) was immersed into sterile water with 0.05% Tween 80. Each treated leaf section was then placed on a Whatman No.1 sterile filter paper for 15 min to remove excessive conidia suspension [74]. Each treated leaf section was transferred into a Petri dish (90 mm diameter, 15 mm depth) lined with moistened Whatman No.1 sterile filter(.Sigma-Aldrich PTY. LTD., Johannesburg, South Africa) After that, ten adult apterous aphids were transferred onto each leaf section using a camel hairbrush and under an optical microscope. Each treatment had six replicates, and each replicate had ten apterous adult aphids. The petri dishes were sealed with parafilm and incubated in the growing chamber at 25 °C and a photoperiod of 12:12 (L:D) h for seven days. The mortality was observed after five days. Insects were considered dead if they remained unresponsive after probing with camel hairbrush. Aphids that died were sterilized by dipping them into 70% ethanol for 10 s and rinsed with sterile distilled water for 1 min. The cadavers were moved to Petri dishes lined with damp filter paper and were incubated at 25 °C in the dark, with 90% relative humidity to promote fungal growth and sporulation. Mycelial outgrowth from on the insect cadaver was an indication that the insect died from the fungus.

### 4.5. Greenhouse Study

This experiment took place at CPUT in the Department of Horticultural Sciences, Bellville Campus, South Africa. Greenhouse’s mean temperature was 27 ± 3 °C, 70% ± 3% relative humidity, and the average light intensity was 31.77 kilo lux. Two weeks old lettuce seedlings were transferred into 15 cm pots containing a substrate mix of 25% silica sand, 25% coco peat, 25% perlite, and 25% vermiculite. One hundred plants were planted into a 15 cm pot, separately. This experiment had four treatments, which were based on varied conidial concentrations, 0 conidia mL^−1^, 1 × 10^6^ conidia mL^−1^, 1 × 10^7^ conidia mL^−1^, and 1 × 10^8^ conidia mL^−1^. Each treatment had twenty-five replicates (*n* = 100). Plants in the fungus treatment were drenched with 100 mL conidial suspension, while control plants were drenched with 100 mL sterile distilled water (0.05% Tween 20). Plants were fed using recommended hydroponics Nutrifeed fertilizer (Starke Ayres Pty. Ltd., Cape Town, South Africa) comprising the following ingredients: N (65 mg kg^−1^), P (27 mg kg^−1^), K (130 mg kg^−1^), Ca (70 mg kg^−1^), Cu (20 mg kg^−1^), Mo (10 mg kg^−1^), Fe (1500 mg kg^−1^), Mg (22 mg kg^−1^), S (75 mg kg^−1^), B (240 mg kg^−1^), Mn (240 mg kg^−1^), and Zn (240 mg kg^−1^). The fertilizer was mixed with sterile distilled water at a concentration of 10g/5000 mL, and 200 mL was added to each plant once a week. Each plant was watered with distilled water twice a week. The data were collected, plant height was measured from the soil surface to the top of the highest leaf, and crown size was measured (widest horizontal width between the leaves). After 21 days post-treatment, fresh leaves were pick-off plants and taken to the laboratory to assess fungal colonization. Twenty-five plants from each of the four treatments (*n* = 100) were screened for fungal colonization. Leaf sections were surfaced sterilized in the following sequence: 0.5% of sodium hypochlorite for two minutes, 70% ethanol for two minutes, and then rinsed with sterile distilled water for 1 min. The sterilized leaf sections were placed on a selective solid agar plates made up of potatoes dextrose agar (PDA) half strength of 19.5 g/1000 mL of sterile water containing 0.04 g streptomycin and 0.02 g ampicillin sodium salt and were incubated at 25 ± 2 °C. After six weeks post-inoculation, plants were uprooted from the pots, and roots length (cm plant^−1^) and fresh weight (g plant^−1^) of plants and roots were measured. Roots were separated from the aerial parts. Sub-samples of lettuce were oven-dried at 35 °C for 168 h, after which the dried plants were weighed (g plant^−1^) of roots and plants. The experiment was repeated twice.

### 4.6. Antioxidants

#### 4.6.1. Sample Material

At the end of the greenhouse experiment, plants were randomly selected based on fungal colonization. Plants were oven-dried at 35 °C for 168 h. The dried plant materials were ground, and the powered material transferred into plastic bags. Three samples representing three replicates were weighed for each treatment (*n* = 12), and 0.1g of powdered plant material was transferred into centrifuge tubes. The samples were extracted with 25 mL of 60% ethanol and placed inside the incubator for 24 h.

#### 4.6.2. Ferric Reducing Antioxidant Power (FRAP)

The ferric reducing antioxidant power assay is similar to the procedure described by Benzie and Strain [75]. This assay is based on the reduction of ferric-tripyridyltriazine complex to its ferrous in the presence of antioxidants. The following reagents were used: 2.5 mL of a 10 mmol/L TPTZ (2,4,6- tripyridyl-s-triazine, Sigma-Aldrich, Johannesburg, South Africa) solution in 40 mmol/L HCl plus 2.5 mL of 20 mmol/L FeCl_3_ and 25 mL of 0.3 mol/L acetate buffer and maintained at pH 3.6 was prepared freshly and warmed at 37 °C. Aliquots of 40 μL of the sample supernatant were mixed with 0.2 mL distilled water and 1.8 mL FRAP reagent. After incubation at 37 °C for 10 min, we employed spectrophotometric method to determine the absorbance of the reaction mixture at 593 nm. The standard solution was 1 mmol/L of FeSO_4_, and the final result was expressed as the concentration of antioxidants having a ferric reducing ability equivalent to 1 mmol/L FeSO_4_. 

#### 4.6.3. Trolox Equivalent Antioxidant Capacity (TEAC)

The scavenging ability of the antioxidants in lettuce was measured using the TEAC method described by Miller et al. [76]. The TEAC value is based on antioxidant’s ability to scavenge the blue-green colored 2,2′-azino-bis-(3-ethylbenzthiazoline-6-sulphonic acid) radical (ABTS^•+^) radical cation relative to the ABTS^•+^ radical cation scavenging ability of the water-soluble vitamin E analogue. 

### 4.7. Tissue Nutrient Content Analysis

After six week-post-inoculation, 12 plants that showed fungal colonization, three from each treatment, were used for the analysis of macronutrients and micronutrients at a commercial laboratory, Bemlab (Pty) Ltd (Strand, Cape Town, South Africa). Before the analyses, the lettuce leaves were washed with teepol solution, followed by rinsing with sterile distilled water, and then drying at 65 °C overnight in an oven. The dried leaves were milled and ashed at 480 °C, shaken up in a 50:50 HCl (50%) solution for extraction through filter paper [77]. The phosphorus (P), potassium (K), calcium (Ca), magnesium (Mg), Sodium (Na), Iron (Fe), manganese (Mn), zinc (Z), Boron (B), copper (Cu), and carbon (C) content of the extracts were analyzed as described by Miller [77]. Total nitrogen (N) content of the leaves was assessed through total combustion in a Leco N-analyser. The unit of the macronutrients was g kg^−1^ while micronutrients were expressed as mg kg^−1^.

### 4.8. Proximate Analysis

#### 4.8.1. Sample Preparation

Briefly, after 30 min of harvest, lettuce plants were frozen at −20 °C and lyophilized. Three plants from each treatment (*n* = 12 for the four treatments) were used for the analysis. The damaged leaves were carefully removed during this preparation. Dried materials were grounded with an ultracentrifuge mill.

#### 4.8.2. Protein Analysis

The method used was adopted from Chikwanha et al. [78]; nitrogen content was analyzed using the method described by Duma of macro-Nitrogen analyzer (LECO^®^ FP528, LECO Corporation, Miami, FL, USA). Total protein content was determined by multiplying the N content by a factor of 6.25. The total protein percentage was converted into g kg^−1^.

#### 4.8.3. Fatty Acid Analysis

The method was adopted from Sukhija and Palmquist [79] with minor adjustment. Fatty acids were analyzed on Agilent 7890A Gas Chromatography—Flame Ionization Detector System (Agilent Technologies, Inc., Santa Clara, CA, USA). The column used was HP88 (100 m × 250 μm, 0.250 μm); the temperature was set at 50 °C hold for 2 min, increase at 5 °C/min to 250 °C, and hold for 15 min. Carrier gas: Nitrogen with a flow rate set at 1.0 mL/min. Injection volume: 1 μL (split; 50:1). The fatty acids were detected by evaluation of their retention times with that of internal standard. The fatty acids that were detected were expressed as mg kg^−1^.

### 4.9. Chlorophyll Content Analysis

The chlorophyll analyses were based on the methods described by Arnon [80] and Rajalakshmi and Banu [81]. Briefly, one gram of freshly harvested plants was ground with 20–40 mL of acetone. It was then centrifuged for 5 min at 5000–10,000 rpm. After that, the supernatant was used for absorbance reading. The absorbance of the solution was read at 645 nm and 663 nm against the solvent (acetone) blank.

#### Estimation of Chlorophyll Content 

The concentrations of total chlorophyll, chlorophyll a, and chlorophyll b were calculated using the following equations [81]

Total Chlorophyll: 20.2(A_645_) + 8.02(A_663_)

Chlorophyll a: 12.7(A_663_) − 2.69(A_645_)

Chlorophyll b: 22.9(A_645_) − 4.68(A_663_)

### 4.10. Statistical Analysis

The data collected were plant height (cm), crown size (cm), roots length (cm), plant dry weight (g), roots dry weight (g), plant fresh weight (g), roots fresh weight (g), and FRAP (Umol AAE/g), TEAC (Umol TE/g), macronutrients (g kg^−1^), micronutrients (mg kg^−1^), protein (mg kg^−1^), and fatty acids (mg kg^−1^), and chlorophyll (Mean ± SE μg g^−1^). The growth parameters’ data of the first and second experiments were pooled since no significant differences were observed when the growth results were compared. The data were then analyzed using one-way ANOVA. The data on tissue colonization were analyzed using the Fisher’s exact test. Dosage mortality response was subjected to Finney’s probit analysis method [82] to obtain the LC_50_. The analyses were performed using the statistical software TIBCO Statistica^®^ 13.3.0 Dell Inc., California, USA. Count mortality data were arcsine square-root transformed and analyzed using one-way analysis of variance (ANOVA). The post hoc Turkey HSD was applied to separate means.

## 5. Conclusions

The *B. bassiana* strain used in this study was pathogenic and successfully colonized the lettuce plants inoculated with its conidia. This study further demonstrated that endophytic entomopathogen (*B. bassiana*) used in this study can be used to agriculturally produce biofortified lettuce—tissue micronutrients, proteins and antioxidant capacity were significantly enhanced by *B. bassiana* inoculation. Remarkably, we found a strong association between tissue C content and antioxidant capacity of lettuce. This study revealed that tissue macronutrient and micronutrient contents can potentially mediate the influence of endophytic fungus on plant growth, antioxidant capacity and chlorophyll content. Future studies should investigate the effects of *B. bassiana* strain (SM 3) on plant primary and secondary metabolite contents and aphid infestations on lettuce.

## Figures and Tables

**Table 1:** The pathogenicity (mean ± SE number of dead insects and (Abbott-corrected percentage mortality), of *Beauveria bassiana* against *Myzus persicae* in the laboratory at five days post-treatment.

Treatments	Mean ± SE Number of Dead Insects and (Abbott-Corrected Percentage Mortality)
Control	0 ± 0
1 × 10^6^ conidia mL^−1^	48 ± 0.50
1 × 10^7^ conidia mL^−1^	66 ± 0.31
1× 10^8^ conidia mL^−1^	78 ± 0.18
**LC_50_** **Fiducial limits (95% CI)**	1.2 × 10^6^ conidia mL^−1^ 1.1 × 10^6^–1.6 × 10^6^ conidia mL^−1^

Means with the same lowercase letters in the column indicates means ± SE are not significantly different using Tukey HSD test at *p* = 0.05 level of significance.

**Table 2:** The effect of *Beauveria bassiana* on root length, plant height and crown size of the plants (*Lactuca sativa*) at varying conidial concentrations (*n* = 200).

Treatments	Roots Length (cm)	Plant Height (cm)	Crown Size (cm)
Control	19.50 ± 0.54 a	15.52 ± 0.10 a	25.94 ± 0.19 a
1 × 10^6^ conidia mL^−1^	19.33 ± 0.47 a	16.02 ± 0.12 b	27.50 ± 0.27 bc
1 × 10^7^ conidia mL^−1^	20.10 ± 0.51 a	16.02 ± 0.12 b	26.96 ± 0.29 b
1 × 10^8^ conidia mL^−1^	20.33 ± 0.38 a	16.04 ± 0.18 b	28.14 ± 0.22 c

The same lowercase letters in the same column indicates means ± SE are not significantly different using Tukey HSD test at *p* = 0.05 level of significance.

**Table 3:** The effect of *Beauveria bassiana* on dry and fresh weights of aerial part and roots of plants (*Lactuca sativa*) exposed to different conidial concentrations (*n* = 120).

Treatments	Roots Fresh Weight (g)	Plant Fresh Weight (g)	Roots Dry Weight(g)	Plant Dry Weight (g)
Control	23.57 ± 0.62 a	58.70 ± 1.60 a	3.18 ± 0.12 a	4.24 ± 0.11 a
1 × 10^6^ conidia mL^−1^	23.12 ± 1.21 a	61.70 ± 1.53 a	3.30 ± 0.06 a	4.49 ± 0.13 a
1 × 10^7^ conidia mL^−1^	20. 48 ± 1.00 a	54.17 ± 2.26 a	3.39 ± 0.11 a	4.61 ± 0.11 a
1 × 10^8^ conidia mL^−1^	21.28 ± 0.95 a	57.65 ± 2.43 a	3.32 ± 0.10 a	4.49 ± 0.18 a

The same lowercase letters in the same column indicates means ± SE are not significantly different using Tukey HSD test at *p* = 0.05 level of significance.

**Table 4:** The effect of *Beauveria bassiana* on the antioxidant capacity of lettuce extracts following exposure of plants (*Lactuca sativa*) to different conidial treatments during cultivation (*n* = 12).

Treatments	Frap (µmol AAE/g) Mean ± SE	TEAC (µmol TE/g) Mean ± SE
Control	86.13 ± 6.35 a	88.92 ± 7.02 a
1 × 10^6^ conidia mL^−1^	46.15 ± 3.61 b	32.17 ± 6.43 b
1 × 10^7^ conidia mL^−1^	55.81 ± 13.15 b	43.14 ± 4.40 b
1 × 10^8^ conidia mL^−1^	89.43 ± 9.10 a	97.97 ± 5.30 a

The same lowercase letters in the same column indicates means ± SE are not significantly different using Tukey HSD test at *p* = 0.05 level of significance.

**Table 5:** Effects of inoculating lettuce plants (*Lactuca sativa*) with different conidial concentrations of *Beauveria bassiana* on tissue macronutrients contents (g kg^−1^) (*n* = 12).

Treatments	C	N	P	K	Ca	Mg	Na
Control	407.36 ± 0.41 a	20.90 ± 0.60 a	4.10 ± 0.60 a	55.33 ± 0.33 a	10.10 ± 0.45 a	4.27 ± 0.15 a	2.43 ± 0.14 a
1 × 10^6^ conidia mL^−1^	365.50 ± 3.36 b	21.37 ± 0.75 a	4.67 ± 0.38 a	60.00 ± 4.16 a	11.67 ± 0.88 a	5.27 ± 0.56 a	2.58 ± 0.19 a
1 × 10^7^ conidia mL^−1^	363.13 ± 1.48 b	22.13 ± 0.47 a	4.53 ± 0.18 a	60.67 ± 0.67 a	10.33 ± 0.33 a	5.20 ± 0.23 a	2.42 ± 0.04 a
1 × 10^8^ conidia mL^−1^	396.33 ± 6.55 a	21.20 ± 0.81 a	4.60 ± 0.30 a	58.33 ± 2.73 a	10.03 ± 0.48 a	4.63 ± 0.20 a	2.44 ± 0.29 a

The same lowercase letters in the same column indicates means ± SE are not significantly different using Tukey HSD test at *p* = 0.05 level of significance.

**Table 6:** Effects of inoculating lettuce plants (*Lactuca sativa*) with different conidial concentrations of *Beauveria bassiana* on tissue micronutrients contents (mg kg^−1^) (*n* = 12).

Treatments	Mn	Fe	Cu	Zn	B
Control	54.73 ± 5.25 a	286.00 ± 17.47 a	2.87 ± 0.33 a	47.27 ± 7.13 a	38.70 ± 1.29 a
1 × 10^6^ conidia mL^−1^	70.63 ± 7.58 ab	439.33 ± 41.91 b	5.00 ± 0.15 b	39.77 ± 3.48 a	45.43 ± 2.57 ab
1 × 10^7^ conidia mL^−1^	81.03 ± 4.39 b	427.33 ± 2.85 b	5.90 ± 0.26 b	38.33 ± 4.99 a	50.27 ± 1.01 b
1 × 10^8^ conidia mL^−1^	72.60 ± 1.99 ab	464.67 ± 34.36 b	3.57 ± 0.35 a	36.10 ± 2.21 a	46.27 ± 3.44 ab

The means ± SE followed by the same lowercase letters column indicates means ± SE are not significantly different using Tukey HSD test at *p* = 0.05 level of significance.

**Table 7:** Effects of inoculating lettuce plants with different conidial concentrations of *Beauveria bassiana* on protein contents (g kg^−1^) (*n* = 12).

Treatments	Protein (g kg^−1^) Mean ± SE
Control	27.87 ± 2.02 ab
1 × 10^6^ conidia mL^−1^	23.20 ± 1.19 a
1 × 10^7^ conidia mL^−1^	32.01 ± 2.25 ab
1 × 10^8^ conidia mL^−1^	34.06 ± 2.26 b

The same lowercase letter in the same column indicates means ± SE are not significantly different using Tukey HSD test at *p* = 0.05 level of significance.

**Table 8:** Influence of *Beauveria bassiana* on proximate composition of *Lactuca sativa* (mg kg^−1^) (*n* = 12).

Treatments	Palmitic Acid Mean ± SE	Linoleic Acid Mean ± SE	Linolenic Acid Mean ± SE	Total Fatty Acids Mean ± SE
Control	366.67 ± 33.33 a	266.67 ± 33.33 a	933.33 ± 166.67 a	1566.67 ± 233.33 a
1 × 10^6^ conidia mL^−1^	466.67 ± 166.67 a	200.00 ± 0.00 a	566.67 ± 33.33 a	1233.33 ± 185.59 a
1 × 10^7^ conidia mL^−1^	300.00 ± 0.00 a	200.00 ± 0.00 a	733.33 ± 33.33 a	1233.00 ± 33.33 a
1 × 10^8^ conidia mL^−1^	400.00 ± 0.00 a	200.00 ± 0.00 a	833.33 ± 33.33 a	1433.33 ± 33.33 a

The same lowercase letter in the same column indicates means ± SE are not significantly different using Tukey HSD test at *p* = 0.05 level of significance. ND denotes not detected.

**Table 9:** Effects of inoculating lettuce plants with different conidial concentrations of *Beauveria bassiana* on total chlorophyll and chlorophyll contents a and b (Mean ± SE µg g^−1^) (*n* = 12).

Treatments	Total Chlorophyll	Chlorophyll a	Chlorophyll b
Control	503.13 ± 36.73 a	368.51 ± 28.31 a	134.75 ± 8.45 a
1 × 10^6^ conidia mL^−1^	488.93 ± 6.71 a	355.16 ± 5.15 a	133.89 ± 1.56 a
1 × 10^7^ conidia mL^−1^	520.84 ± 25.97 a	381.12 ± 17.63 a	139.85 ± 8.38 a
1 × 10^8^ conidia mL^−1^	586.51 ± 31.21 a	425.39 ± 22.37 a	161.26 ± 8.82 a

Means with the same lowercase letters in the same column are not significantly different following comparison using the Tukey HSD at *p* = 0.05 level of significance.

## Data Availability

Data is contained within the article.

## Supplementary Materials

The following are available online at https://www.mdpi.com/article/10.3390/plants10061178/s1, Figure S1. Mycelia outgrowth from leaf sections demonstrating successful colonization of tissues by the endophytic fun-gus *Beauveria bassiana*. Table S1. Correlation between tissue nutrients and anti-oxidant capacity (FRAP and TEAC).
