# Supplementary material for: Evaluating the Endophytic Activities of Beauveria bassiana on the Physiology, Growth, and Antioxidant Activities of Extracts of Lettuce (Lactuca sativa L.)
Source: Plants (Basel). 2021 Jun 9;10(6):1178. doi: 10.3390/plants10061178 (PMC8229626; doi:10.3390/plants10061178)
Supplement: Supplementary file 1 [file plants-10-01178-s001.zip › plants-1187600-supplementary.pdf]

Table S1. Correlation between tissue nutrients and anti-oxidant capacity (FRAP and TEAC).

Nutrients	FRAP	TEAC
Mn	$R^2 = 0.27$<br>y =0.26x + 87.90	$R^2 = 0.25$<br>y = -0.17x + 80.78
Fe	$R^2 = 0.13$<br>y = -1.32x +495	$R^2 = 0.10$<br>y = -0.76x + 452.76
Cu	$R^2 = 0.741$<br>y = -0.05x + 8.11	$R^2 = 0.75$<br>y = -0.036x + 6.71
B	$R^2 = 0.27$<br>y = -0.12x + 53.14	$R^2 = 0.26$<br>y = -0.07x + 50.03
C	$R^2 = 0.88$<br>y = 0.96x + 316.41	$R^2 = 0.87$<br>y = 0.63x + 341.66

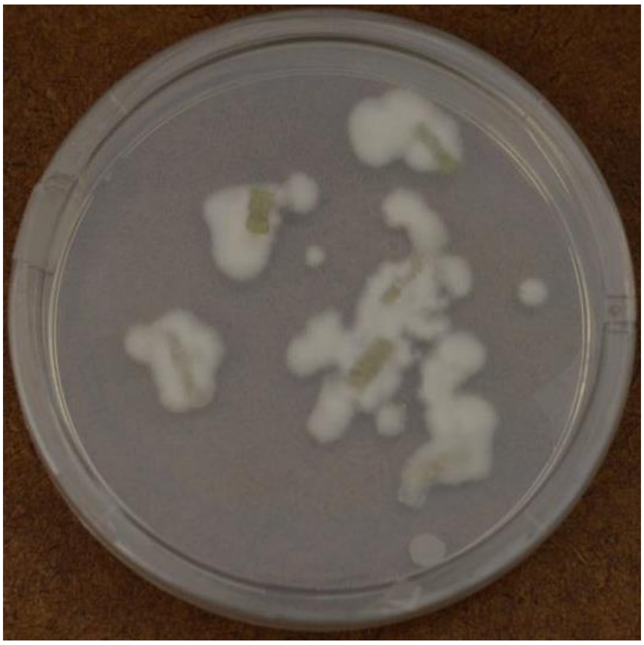

Figure S1. Mycelia outgrowth from leaf sections demonstrating successful colonization of tissues by the endophytic fungus *Beauveria bassiana.*
